# Glucocerebrosidase activity, cathepsin D and monomeric α-synuclein interactions in a stem cell derived neuronal model of a PD associated *GBA1* mutation

**DOI:** 10.1016/j.nbd.2019.104620

**Published:** 2020-02

**Authors:** Shi-yu Yang, Matthew Gegg, David Chau, Anthony Schapira

**Affiliations:** Department of Clinical and Movement Neurosciences, UCL Queen Square Institute of Neurology, London, UK

**Keywords:** Parkinson's disease, Glucocerebrosidase, Monomeric α-Synuclein, Cathepsin D, Cerezyme, Ambroxol, PD, Parkinson's disease, GBA, Glucocerebrosidase gene, GCase, Glucocerebrosidase enzyme, CTSD, cathepsin D, CTSB, Cathepsin B, NCSC, Neural crest stem cell, ABX, ambroxol, LAMP1, Lysosomal-associated membrane protein 1

## Abstract

The presence of *GBA1* gene mutations increases risk for Parkinson's disease (PD), but the pathogenic mechanisms of *GBA1* associated PD remain unknown. Given that impaired α-synuclein turnover is a hallmark of PD pathogenesis and cathepsin D is a key enzyme involved in α-synuclein degradation in neuronal cells, we have examined the relationship of glucocerebrosidase (GCase), cathepsin D and monomeric α-synuclein in human neural crest stem cell derived dopaminergic neurons. We found that normal activity of GCase is necessary for cathepsin D to perform its function of monomeric α-synuclein removal from neurons. *GBA1* mutations lead to a lower level of cathepsin D protein and activity, and higher level of monomeric α-synuclein in neurons. When *GBA1* mutant neurons were treated with GCase replacement or chaperone therapy; cathepsin D protein levels and activity were restored, and monomeric α-synuclein decreased. When cathepsin D was inhibited, GCase replacement failed to reduce monomeric α-synuclein levels in *GBA1* mutant neurons. These data indicate that *GBA1* gene mutations increase monomeric α-synuclein levels via an effect on lysosomal cathepsin D in neurons.

## Highlights

• Cathepsin D protein and activity decreased in GBA mutation-associated PD neurons.
• α-synuclein protein level increased in GBA mutation-associated PD neurons.
• GBA enzyme replacement treatment increased cathepsin D, decreased α-synuclein levels.
• GBA enzyme chaperone treatment increased cathepsin D, decreased α-synuclein levels.
• The influence of GBA enzyme replacement on α-synuclein mediated through cathepsin D.

## 1 Introduction

Glucocerebrosidase gene (*GBA1*) mutations are recognised as the most important genetic risk factor for Parkinson's disease (PD) (Balestrino and Schapira, 2018; Sidransky et al., 2009). Approximately 10–25% of PD patients carry *GBA1* mutations, their presence increases the risk for PD in any one individual by up to 20 times, depending on ethnicity (Zhao et al., 2016). PD patients with *GBA1* mutations generally have an earlier age of onset (Beavan et al., 2015; Brockmann et al., 2011; Neumann et al., 2009); glucocerebrosidase (GCase) activity is reduced in the substantia nigra of PD brain, particularly in those with *GBA1* mutations (Gegg et al., 2012). GCase is a lysosomal housekeeping enzyme which catalyses glucosylceramide and glucosylsphingosine breakdown into glucose and ceramide or sphingosine respectively. Homozygous mutations in the *GBA1* gene cause the autosomal recessive lysosomal storage disorder Gaucher disease (GD) with the accumulation of glucosylceramide. Both homozygous and heterozygous *GBA1* mutation carriers have a similar risk for the development of PD, but no accumulation of GCase substrate has yet been observed in PD brains with *GBA1* mutations (Gegg et al., 2015; Neumann et al., 2009).

Aggregation of *α*-synuclein protein in the form of Lewy bodies and Lewy neurites in neurons in the substantia nigra, cerebral cortex and hippocampus are the neuropathological hallmarks of PD. Monomeric *α*-synuclein is a small protein (14 kDa) highly expressed in the brain; its biological functions are not completely understood, but it is thought to be involved in vesicular release and transport. *α*-synuclein directly binds to vesicle-associated membrane proteins (Burre et al., 2010; Siebert et al., 2014) and regulates synaptic vesicle mobilization at nerve terminals (Cabin et al., 2002). *α*-synuclein may be involved in the regulation of synaptic vesicle clustering and the release of neurotransmitters (Siebert et al., 2014). Cathepsin D (CTSD) is a lysosomal aspartic *endo*-protease which is involved in the degradation of α-synuclein and generation of its carboxy-terminal truncated species (Sevlever et al., 2008). The presence of anionic phospholipids are crucial for CTSD to cleave throughout the *α*-synuclein sequence (McGlinchey and Lee, 2015). CTSD levels influence α-synuclein processing, aggregation and toxicity; its deficiency leads to intracellular accumulation of α-synuclein in mice, sheep and human infant brain (Cullen et al., 2009).

There is evidence for a reciprocal interaction between GCase and α-synuclein levels. For example, reduced GCase activity promoted α- synuclein aggregation in vitro and in vivo (Mazzulli et al., 2011; Sardi et al., 2011); a GCase inhibitor increased α- synuclein levels in vitro and aggregation in the substantia nigra in vivo in mice (Cleeter et al., 2013; Rocha et al., 2015b). In human stem cell derived dopaminergic neuronal models, heterozygous *GBA1* mutations reduced GCase protein and activity, and increased monomeric α-synuclein levels (Schondorf et al., 2014; Yang et al., 2017). Treating with the GCase chaperone ambroxol (ABX), which increases GCase protein levels and activity, or GCase enzyme replacement can decrease monomeric α-synuclein levels in human dopaminergic neurons (Yang et al., 2017).

Ceramide, the product of the GCase enzymatic reaction, is an activator of CTSD (Heinrich et al., 2000). It can specifically bind CTSD and increase its stability and proteolytic activity (Heinrich et al., 1999). *GBA1* mutations reduce GCase activities which in turn would decrease ceramide levels in lysosomes and so could reduce CTSD protein levels and activities. This in turn would result in increased levels of α-synuclein. CTSD protein and activity are reduced in the frontal cortex of PD and Lewy body dementia brains with *GBA1* mutation (Kurzawa-Akanbi et al., 2012). We examined the relationship between *GBA1* mutations, cathepsin D (pro- and mature CTSD) and monomeric α-synuclein levels in neural crest stem cells (NCSC)-derived dopaminergic neurons from heterozygous *GBA1* mutation carriers with PD, and found reduced levels of CTSD (pro- and mature CTSD) protein and activity; and higher levels of monomeric α-synuclein. Replacement of the mutant GCase with recombinant GCase increased CTSD (pro- and mature CTSD) protein level and its activity; decreased monomeric α-synuclein levels in dopaminergic neurons. These results indicate that increased levels of monomeric α-synuclein in *GBA1* mutant neurons are at least in part mediated through reduced CTSD proteins and its activity.

## 2 Material and methods

### 2.1 Subjects and sample collection

Six individual subjects (WT/WT healthy and WT/N370S PD) were used in the study, written informed consent was obtained before the samples were collected. The previous published procedures (Yang et al., 2017) were followed for the collection of samples and preparation.

### 2.2 Growth medium

DMEM, (High Glucose, Gutamax, Life technologies) supplemented with foetal bovine serum (10%), Sodium Pyruvate (1 mM), Uridine (50 μg/ml), Penicillin (50 units/ml), Streptomycin (50 μg/ml), Fungizone (Amphotericin B, 1.25 μg/ml).

### 2.3 Neuronal induction medium (first 10 days of differentiation)

Neurobasal medium supplemented with B-27 supplement (1×), Recombinant Human Sonic Hedgehog (250 ng/ml), Recombinant Human/Mouse FGF-8b (100 ng/ml), Recombinant Human FGF basic (50 ng/ml), Pen strep (50 units/ml) and Fungizone (Amphotericin B, 1.25 mg/ml).

### 2.4 Neuronal maturation medium (11–40 days of differentiation)

Neurobasal medium supplemented with B-27 supplement (1×), Recombinant Human Sonic Hedgehog (250 ng/ml), Recombinant Human/Mouse FGF-8b (100 ng/ml), Recombinant Human FGF basic (100 ng/ml), Recombinant Human/Mouse/Rat/Canine/Equine BDNF (50 ng/ml), Pen strep (50 units/ml) and Fungizone (Amphotericin B, 1.25 μg/ml).

### 2.5 Growth factors

Recombinant human sonic hedgehog (c24II), Recombinant human/mouse FGF-8b, Recombinant human FGF basic (146aa) and Recombinant human/mouse/rat/canine/equine BDNF were purchased from R and D Systems.

### 2.6 Dopaminergic neuronal differentiation

NCSC were detached with accutase solution and the accutase was neutralized by the addition of growth medium. Cells were seeded in fibronectin coated 6 well plates at a density of 2.4 × 10^4^ cells/well (for immunocytochemistry assay, cells were seeded onto coverslips coated with fibronectin within a 6-well plate) with growth medium. After 24 h of seeding, the growth medium was removed from the well; cells were washed once with neurobasal medium and then cultured with neuronal induction medium. The cells were cultured with 5% CO_2_/ 95% air for 10 days for neuronal induction. Following neuronal induction, the neuronal induction medium was replaced with neuronal maturation medium. The cells were cultured with 5% CO_2_/ 95% air for 30 days. The volume of neuronal maturation medium was 2 ml/well (6-well plate). The medium was changed with the freshly made neuronal maturation medium every 5 days during maturation.

### 2.7 Characterisation of differentiated neurons

Western blotting and immunochemistry was carried out to examine the expression of tyrosine hydroxylase (TH) and the nuclear receptor related 1 protein (Nurr1) in the differentiated cells. TH and Nurr1 are two specific dopaminergic neuronal marks.

### 2.8 Postmortem brain material

Brain samples were received from the Queen Square Brain Bank for Neurological Disorders, UCL Institute of Neurology. The donation program and protocols have ethical approval for research by the NRES Committee London—Central and tissue is stored for research under a license issued by the Human Tissue Authority (No. 12198). Samples analysed were three controls (mean age 74 ± 11 years; post mortem delay 48.7 ± 14.6 h), three PD brains with N370S/WT *GBA1* mutations (mean age 86 ± 3 years; post mortem delay 54.4 ± 3.6 h) and three sporadic PD brains (mean age 67 ± 1 years; post mortem delay 38.9 ± 2.3 h).

### 2.9 GCase activity assays

Cell pellets were re-suspended in water and sonicated in a water-bath sonicator for 1 min. GCase activity was determined in cell lysate of about 1 μg protein with previous reported method (Cleeter et al., 2013).Enzyme activities were calculated by subtracting the background fluorescence from the mean fluorescence measured for a given cell lysate, then dividing by the standard to calculate the nmol/h/ml activity. This result was then divided by the total protein concentration, as determined using the BCA method, to calculate the enzymatic activity in nmol/h/mg.

### 2.10 CTSD activity assay

The neuronal CTSD activity was determined with a CTSD activity fluorometric assay kit following the manufacturer's instructions.

### 2.11 Immunochemistry

Cells were washed with PBS two times, each wash lasting for 5 min. Cells were fixed with 4% paraformaldehyde for 15 min at room temperature, subsequently permeabilized with 0.25% Triton X-100 for 15 min. Following three times washing, cells were blocked with 10% goat serum in PBS for 30 min and incubated with primary antibodies overnight at 4 °C (Table 1). The appropriate secondary antibodies conjugated with fluorescein were used to visualize the positive stained cells.

**Table 1:** List of antibodies.

Antibodies	Dilution	Applications	Source	Cat. No
Nurr 1	1:200	ICC	abcam	ab55769
Tyrosine Hydroxylase	1:1000	ICC/WB	abcam	ab6211
β –III tubulin	1:500	ICC/WB	abcam	ab18207
GBA	1:1500	ICC/WB	Calbichem	AP1140
β-actin	1:5000	WB	abcam	ab8227
Pro-CTSD (46kD)	1:1000	WB	abcam	ab134169
CTSD (28kD)	1:1000	WB	abcam	ab75852
CTSB	1:1000	WB	abcam	ab125067
α-synuclein	1:750	WB	abcam	Ab1903
LAMP1	1:4000	WB	Novus biologicals	NBP2–25155

### 2.12 Western blotting

Cells were harvested, washed with PBS, and processed as previously described (McNeill et al., 2014). Proteins were extracted using urea/SDS buffer (8 M urea, 2% SDS, 10 mM Tris) containing protease inhibitors on ice. To limit the effect of DNA content in the protein solution, the extraction was incubated with DNase at 37^o^ C for 1 h to remove DNA. In total, 30 μg of the protein was loaded on a 4–12% Bis-Tris Gel (NuPAGE, Invitrogen) and transferred to a polyvinylidene difluoride membrane (Millipore, Watford, England). The membrane was incubated with primary antibodies in 5% milk PBST at 4 °C overnight (Table 1). Following 3 times washing, the membrane was incubated with the appropriate secondary antibodies conjugated with horseradish peroxidase for 1 h at room temperature. The bands were visualized with western ECL substrate kit (Bio-Rad). Analyses were carried out using Image Lab software (BioRad).

### 2.13 Inhibition of Cathepsin D

Following 42 days of dopaminergic neuronal differentiation, the neuronal maturation medium was removed from each well. 2 ml of fresh neuronal maturation medium containing Pepstatin A (1 μM) was added to each well (6-well plate). The medium containing Pepstatin A was changed every 2 days. The treatment lasted for 6 days. After treatment the cells were washed twice with PBS and harvested with accutase. Cell pellet was kept at -80 °C.

### 2.14 Cerezyme treatment

Following dopaminergic neuronal differentiation, old neuronal maturation medium was removed. 2 ml of freshly made neuronal maturation medium including Cerezyme (0.27 U/ml) was added to each well of 6-well plate. The neuronal maturation medium including Cerezyme was changed every 2 days, the total treatment time was 6 days. After treatment, neurons was washed with PBS and harvested with accutase, cell pellets were kept at -80 °C.

### 2.15 Cerezyme treatment with inhibition of Cathepsin D

Following 42 days of dopaminergic neuronal differentiation, the neuronal maturation medium was removed from each well. 2 ml of freshly neuronal maturation medium containing pharmaceutical Cerezyme (2.7Units/ml) and Pepstatin A (1 μM) was added to each well (6-well plate). The medium was changed every 2 days. The treatment lasted for 6 days. After treatment the cells were washed twice with PBS and harvested with accutase. Cell pellets were kept at -80 °C.

### 2.16 Ambroxol treatment

Following 42 days of dopaminergic neuronal differentiation, the neuronal maturation medium was removed from each well. 2 ml of fresh neuronal maturation medium containing ABX (30 μM) was added to each well (6-well plate). The medium was changed every 2 days. The treatment lasted for 6 days. After treatment the cells were washed twice with PBS and harvested with accutase. Cells pellet were kept at -80 °C.

### 2.17 Statistical analysis

Data are expressed as mean ± SEM and statistical significance between groups determined by one-way ANOVA followed by the two-tailed *t*-Test. A *p* value of <0.05 was considered as significantly different. All data were analysed by GraphPad Prism 6 statistical software.

## 3 Results

### 3.1 Dopaminergic neuronal differentiation of neural crest stem cell derived from normal (WT/WT) and *GBA1* mutant (N370S/WT) subjects

**Fig. 1:**
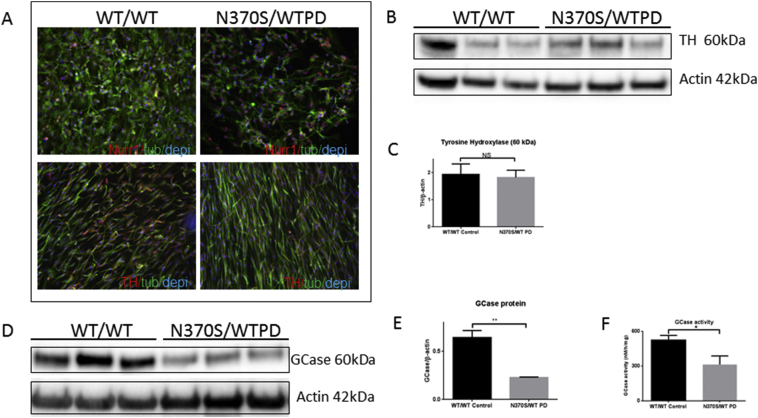
Human neural crest stem cells were differentiated into dopaminergic neurons as previously reported for 42 days (Yang et al., 2017). The midbrain dopaminergic neuronal markers nuclear receptor-related protein 1 (Nurr1) and Tyrosine hydroxylase (TH) were detected against β-III tubulin and DAPI (A). The expression of TH protein was compared between wild type (WT/WT) and *GBA1* mutant (N370S/WT) PD neurons (B and C). GBA protein was compared between wild type (WT/WT) and *GBA1* mutation (N370S/WT) PD neurons (D and E). GCase activity compared between wild type (WT/WT) and *GBA1* mutant (N370S/WT) PD neurons (F).

Neural crest stem cells derived from 6 individuals (3 WT/WT and 3 N370S/WT PD) were differentiated to dopaminergic neurons for 42 days (Yang et al., 2017). The differentiated cells were characterised by immunochemistry with the midbrain dopaminergic neuronal biomarkers, nuclear receptor related 1 protein (NURR1) and tyrosine hydroxylase (TH) (Fig. 1A). 35–50% of differentiated cells stained positively with TH. The expression level of TH was examined by western blotting (Fig. 1 B); TH protein levels were not significantly different between control (WT/WT) and *GBA1* mutant (N370S/WT) PD neurons (Fig. 1C). GBA protein levels were assessed by western blotting, the results (Fig. 1 D, E and F) showed both GBA protein and activity were significantly decreased in *GBA1* mutant (N370S/WT) PD neurons, in agreement with the previous findings (Yang et al., 2017).

### 3.2 *GBA1* mutant (N370S/WT) PD neurons had lower levels of CTSD protein with reduced enzymatic activity

**Fig. 2:**
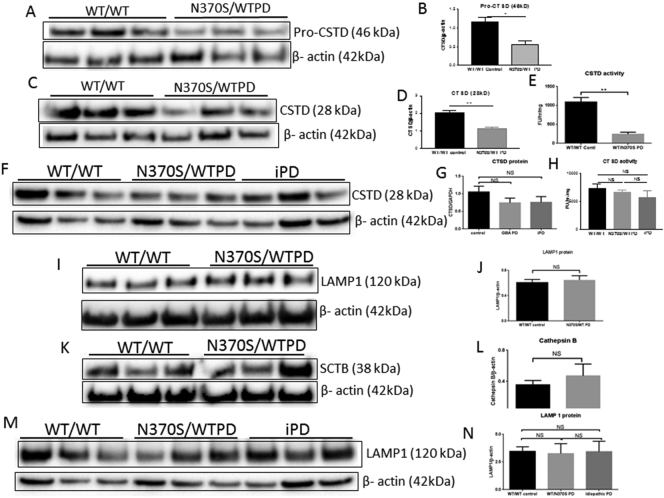
Pro-Cathepsin D (CSTD, 46kD; A and B), mature Cathepsin D (CSTD, 28kD) (C and D) and CSTD activity (E) in wild type (WT/WT) and *GBA1* mutant (N370 s/WT) PD neurons. Putamen mature CSTD and activity in control (WT/WT), *GBA1* mutant (N370S/WT) PD and idiopathic PD patients' brain (F—H). LAMP1 (I and J) and Cathepsin B (SCTB, K and L) protein in wild type (WT/WT) and *GBA1* mutant (N370S/WT) PD neurons. Putamen LAMP1 protein in wild type (WT/WT), *GBA1* mutant (N370S/WT) PD and idiopathic PD patients' brain (M and N).

CTSD (pro and mature) proteins and CTSD activity were determined with Western blotting and a fluorometric assay kit respectively. Pro (46 KDa) and mature (28 KDa) CTSD protein levels and CTSD activity were decreased in *GBA1* mutant (N370S/WT) associated PD neurons, compared with control subjects (Fig. 2 A-E).

We examined mature CTSD in putamen isolated from *GBA1* mutant (N370S/WT) PD, idiopathic PD and matched control brain (*n* = 3), and found the CTSD protein level and activity decreased in *GBA1* mutant (N370S/WT) PD and idiopathic PD patients' putamen (Fig. 2 F—H), but did not reach significance. Lysosomal-associated membrane protein (LAMP-1), a protein responsible for maintaining lysosomal integrity and content was not significantly different between wild type and *GBA*1 mutant neurons (Fig. 2I and J), indicating the *GBA*1 mutation (N370S/WT) did not cause lysosome depletion. Similarly, levels of another lysosomal enzyme Cathepsin B (CTSB) were not affected (Fig. 2K and L). The level of LAMP-1 protein in the putamen tissues was also unchanged (Fig. 2M and N). In summary both pro and mature CTSD protein and its activity was significantly decreased in *GBA1* (WT/N370S) mutant PD stem cell-derived neurons, without any change in lysosome content.

### 3.3 Inhibition of CTSD induced higher level of monomeric α-synuclein in *GBA1* mutant (N370S/WT) PD neurons

**Fig. 3:**
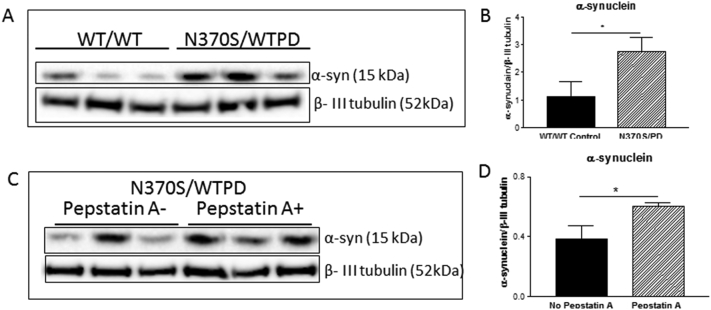
Monomeric α-synuclein protein levels in wild type (WT/WT) and *GBA1* mutant (N370S/WT) PD dopaminergic neurons (A and B). Monomeric α-synuclein protein leve;s in Pepstatin A (a CTSD inhibitor) treated and untreated *GBA1* mutant (N370 s/WT) PD neurons (C and D).

The level of monomeric α-synuclein protein was compared between *GBA1* mutant (WT/N370S) PD and normal (WT/WT) neurons. *GBA1* mutant (WT/N370S) PD neurons had 48% more monomeric *α*-synuclein compared with control neurons (Fig. 3 A and B). Inhibition of CTSD activity in the *GBA1* mutant (WT/N370S) PD neurons with Pepstatin A caused a further significant increase in monomeric *α*-synuclein levels (Fig. 3 C and D). There was no evidence of higher molecular weight bands specific to *α*-synuclein in any of the groups.

### 3.4 GCase replacement treatment restored CTSD level and decreased monomeric α-synuclein in *GBA1* mutant dopaminergic neurons

**Fig. 4:**
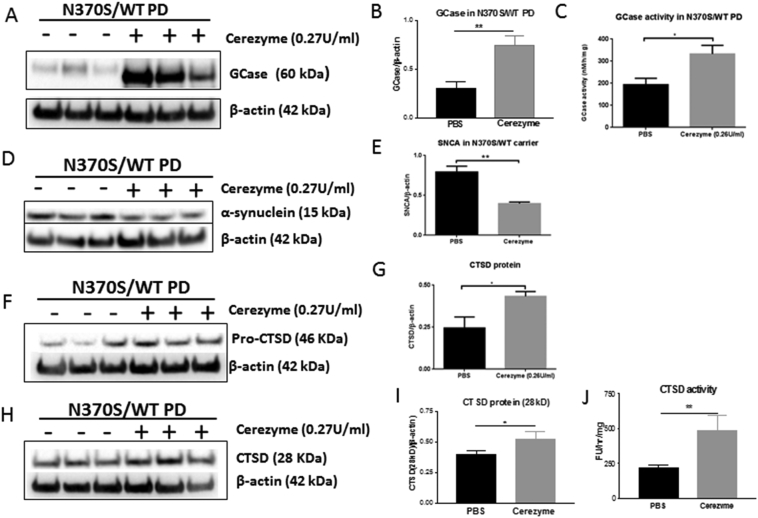
GCase protein and activity in Cerezyme treated and untreated *GBA1* mutant (N370S/WT) PD neurons (A-C). Monomeric α-synuclein protein in Cerezyme treated and untreated *GBA1* mutant (N370 s/WT) PD neurons (D and E). Pro-CTSD protein (46kD) in Cerezyme treated and untreated *GBA1* mutant (N370 s/WT) PD neurons (F and G). Mature-CTSD protein (H and I) and activity in in Cerezyme treated and untreated *GBA1* mutant (N370S/WT) PD neurons (H-J).

Recombinant GCase (Cerezyme) has been used as a long-term enzyme replacement therapy for type 1 Gaucher disease patients. We treated the *GBA1* mutant neurons with Cerezyme for 6 days. Cerezyme was taken up by neurons and increased both GCase protein levels (5.5 fold) and activity (1.7 fold) (Fig. 4 A-C). The GCase replacement by Cerezyme significantly reduced monomeric α-synuclein protein levels by 26% (Fig. 4 D and E) and increased both the pro and mature CTSD protein levels (Fig. 4 F—I) in *GBA1* mutation (N370S/WT) PD neurons. CTSD activity was also significantly increased following GCase replacement treatment (Fig. 4 J).

### 3.5 Ambroxol treatment increased GCase and CTSD proteins and decreased monomeric α-synuclein in *GBA1* mutant PD neurons

**Fig. 5:**
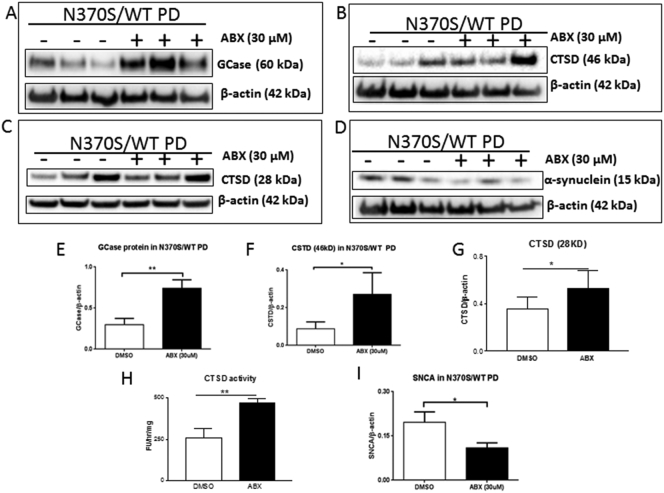
GBA protein in Ambroxol treated and untreated *GBA1* mutant (N370S/WT) PD neurons (A and E). Pro CTSD (46kD) protein in Ambroxol treated and untreated *GBA1* mutant (N370S/WT) PD neurons (B and F). Mature CTSD (28kD) protein and activity in Ambroxol treated and untreated *GBA1* mutant (N370S/WT) PD neurons (C, G and H). Monomeric α-synuclein protein in Ambroxol treated and untreated *GBA1* mutant (N370S/WT) PD neurons (D and I).

Ambroxol (ABX) is a GCase small molecule chaperone and has been reported to increase GBA protein and activity in dopaminergic neurons (Yang et al., 2017), fibroblasts (McNeill et al., 2014), Drosophila (Sanchez-Martinez et al., 2016), mouse midbrain (Migdalska-Richards et al., 2016) and non-human primate brain (Migdalska-Richards et al., 2017).We treated *GBA1* mutant (WT/N370S) dopaminergic neurons with ABX. Following 6 days treatment GBA protein increased 149% in the treated neurons compared with untreated neurons (Fig. 5 A and E), confirming the previous report (Yang et al., 2017). Following ABX treatment, pro-CTSD protein significantly increased 167% (Fig. 5 B and F), mature CTSD protein increase 25% (Fig. 5C and G), CTSD activity increased 88% (Fig. 5 H) and monomeric α-synuclein protein decreased 32% compared with untreated neurons (Fig. 5 D and I). These data, together with the GCase replacement data, suggest that manipulation of GBA protein and its activity enhances the clearance of monomeric α-synuclein in human dopaminergic neuronal cells through regulation of lysosomal CTSD in neurons.

### 3.6 Relationship between GBA, CTSD and monomeric α-synuclein in human neuronal cells

**Fig. 6:**
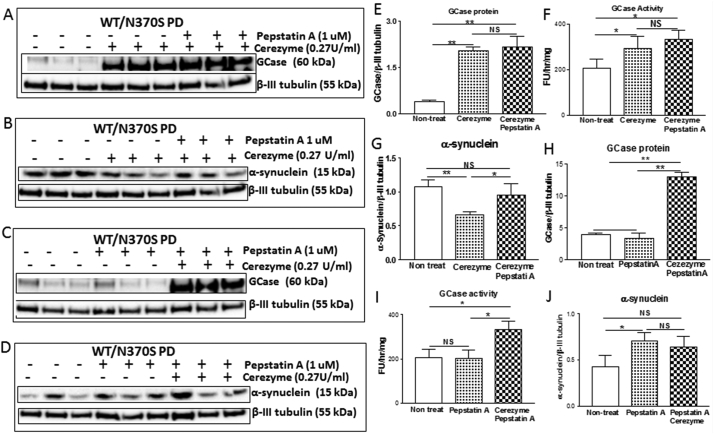
GCase protein and activity in Cerezyme treated (with or without Pepstatin A, a CTSD inhibitor) and untreated *GBA1* mutant (N370S/WT) PD neurons (A, E and F). Monomeric α-synuclein protein in Cerezyme treated (with or without Pepstatin A, a CTSD inhibitor) and untreated *GBA1* mutant (N370S/WT) PD neurons (B and G). GCase protein and activity in Cerezyme treated, Pepstatin A treated and untreated *GBA1* mutant (N370S/WT) PD neurons (C, H and I). Monomeric α-synuclein protein in Cerezyme treated, Pepstatin A treated and untreated *GBA1* mutant (N370S/WT) PD neurons (D and J).

In order to investigate the relationship between GBA, CTSD and monomeric α-synuclein in dopaminergic neurons, we carried out two independent parallel experiments (Fig. 6). In the first experiment (Fig. 6 A-G) we examined whether GCase replacement affects monomeric α-synuclein level in WT/N370SPD dopaminergic neurons and whether this effect is related to CTSD activity. We found that the GCase replacement increased GCase protein levels and activity (Fig. 6 A, E, F). The cerezyme treatment significantly decreased monomeric α-synuclein (Fig. 6 B, G). It is interesting to note that monomeric α-synuclein protein levels did not change compared to untreated neurons when neurons had been treated with both cerezyme and Pepstatin A (a CTSD inhibitor) (Fig. 6 B, G). In the second experiment we examined whether CTSD activity affects monomeric α-synuclein levels in WT/N370S PD dopaminergic neurons and whether these effects are influenced by GCase replacement. Inhibition of CTSD induced a significantly higher level of monomeric α-synuclein (Fig. 6 D, J), the higher level of monomeric α-synuclein was not influenced by GCase protein levels (Fig. 6 C, H) and GCase activity (Fig. 6I). Together these data suggested GCase replacement reduced monomeric α-synuclein level in WT/N370SPD dopaminergic neurons via CTSD activity.

## 4 Discussion

We used a human neural crest stem cell derived dopaminergic neuronal model to investigate pathogenic pathways of *GBA1* mutation associated PD. We found the monomeric α-synuclein protein level was significantly increased in these cells. CTSD protein and enzymatic activity was significantly reduced in the *GBA*1 mutant dopaminergic neurons compared with wild type controls. After recombinant GCase was used to restore GCase activity in *GBA1* mutant neurons, the level of pro- and mature CTSD proteins and activity increased significantly and monomeric α-synuclein levels declined. ABX significantly increased GCase activity in *GBA1* mutant neurons and also increased both pro-and mature CTSD proteins and activity with a corresponding reduction in monomeric α-synuclein levels.

Potential mechanisms that may mediate the effects of *GBA1* mutations include impaired lysosomal function and the unfolded protein response (Fernandes et al., 2016; Mazzulli et al., 2011; Schondorf et al., 2014). Inhibition of macroautophagy flux, and the decreased fusion of autophagosomes with lysosomes has been reported to be coincident with increased monomeric α-synuclein accumulation in GCase-deficient cells (Fernandes et al., 2016; Kilpatrick et al., 2016; Magalhaes et al., 2016; Schondorf et al., 2014; Yang et al., 2017). Decreased CTSD activity has been reported in brains of PD and dementia with Lewy body patients with and without *GBA1* mutation, and correlated with both reduced GCase activity and *GBA1* gene expression (Moors et al., 2019). The same trend in CTSD activity was also reported in CSF of patients with and without GBA1 mutations (Parnetti et al., 2017).

CTSD, an aspartyl protease, is a lysosomal enzyme responsible for the degradation of α-synuclein and generation of its carboxy-terminally truncated species in lysosomes (Sevlever et al., 2008). CTSD expression level affects α-synuclein processing, aggregation and toxicity; its deficiency leads to intracellular accumulation of α-synuclein in mice, sheep and human brain (Cullen et al., 2009). Pharmacological inhibition of CTSD, or introduction of catalytically inactive mutant CTSD to SH-SY5Y cells, an in vitro cell model of dopaminergic neurons, resulted in accumulation of endogenous α-synuclein (Crabtree et al., 2014). α-synuclein associates with negatively charged membranes in forming α-helical structures (Davidson et al., 1998). In the presence of anionic phospholipids, more N-terminal cleavage sites of lipid-bound α-synuclein were exposed, which allows CTSD effectively to cleave α-synuclein in the lysosome (McGlinchey and Lee, 2015). Phospholipids are important in the degradation of α-synuclein. As lipid metabolism changes with age (Rappley et al., 2009) or with disease (Fonteh et al., 2006), it is possible that subtle alterations in phospholipid membrane composition could impact the membrane binding of α-synuclein and its proteolysis; thereby enhancing α-synuclein accumulation in the cytosol. GCase also interacts with anionic phospholipid containing membranes in lysosomes. A study has investigated the relationship between GCase and its activator (saposin C) within anionic phospholipid-containing membrane. In normal anionic phospholipid membranes both normal GCase and mutant (N370S/WT) GCase are able to execute their activity. However, when the amount of anionic phospholipids in the membrane was reduced by approximately 20%, saposin C was still able to activate the normal GCase, but not to activate the mutant (N370S/WT) GCase (Salvioli et al., 2005).

Ceramide is a proteolytic product of GCase and is composed of sphingosine and a fatty acid, which can directly interact with CTSD resulting in autocatalytic proteolysis of the 52 kDa pre-pro cathepsin D to form the enzymatically active 48/32 kDa isoforms of CTSD in the lysosome (Heinrich et al., 1999). Ceramide acts as a lipid second messenger to bind and induce CTSD proteolytic activity (Heinrich et al., 2000). The reduced GCase enzymatic activity of the *GBA1* mutation (WT/N370S) PD neurons would be expected to lower levels of ceramide, which would in turn lead to a lower level of CSTD protein with lower enzymatic activity. This is consistent with our finding of lower levels of CSTD protein and activity in *GBA1* mutation (WT/N370S) neurons (Fig. 2 A, B and C). The lower level of CSTD protein and activity would cause monomeric α-synuclein protein accumulation, as shown in the *GBA1* mutant neurons (Fig. 3 A and B). The importance of CTSD in mediating the increased monomeric α-synuclein associated with *GBA1* mutation is confirmed by the failure of enzyme replacement with cerezyme to lower α-synuclein levels in the presence of the CTSD inhibitor.

The manipulation of the GBA pathway, in particular to enhance GCase activity and lower α-synuclein levels, has attracted attention as a means to slow the progression of pathology in PD (Schapira et al., 2014). There are several potential ways in which this can be achieved. Possibly the simplest would be enzyme replacement, as for GD. However, cerezyme, the replacement used for GD, does not cross the blood brain barrier. Alternative strategies include GBA overexpression by AAV and this has proven effective in rodents (Rocha et al., 2015a; Sardi et al., 2011) and is feasible in humans. Manipulation of *GBA1* gene function and expression could also increase GCase levels and activity. The use of GCase chaperones to enhance GCase has proven successful in several in vitro and in vivo models (Magalhaes et al., 2018; Migdalska-Richards et al., 2017; Sanchez-Martinez et al., 2016; Yang et al., 2017). ABX has been the most studied in this context and increases GCase activity through a chaperone effect, but also by increasing TFEB activity and general lysosomal function, including increasing CTSD (Magalhaes et al., 2018). This drug is currently in clinical trials for PD (AiM-PD; ClinicalTrials.govIdentifier:NCT02941822.) and PD dementia (ClinicalTrials.govIdentifier:NCT02914366). An alternative and potentially complementary strategy is the use of substrate inhibitor to lower glucosylsphingosine, this approach is in use in GD and is currently being evaluated in PD (Moves-PD; ClinicalTrials.govIdentifier:NCT02906020).

The results of the present study add CTSD as an additional potential target for manipulating the GBA pathway to lower monomeric α-synuclein levels in PD.

## Declaration of Competing Interest

The authors declare no competing financial interest.
